# The Diagnosis of Prostate Cancer

**DOI:** 10.1120/jacmp.v17i6.6676

**Published:** 2016-11-08

**Authors:** Robert J. Schulz

**Affiliations:** ^1^ Yale University New Haven CT

To the Editor:

Based upon my own experience, I suspect that the average radiation oncologist knows more about the physics of his specialty than physicists know about cancer. As we are physicists *in medicine* it would seem reasonable that we be moderately conversant about this disease. Although far from proficient, over the past few years I've had occasions to review how prostate cancer (CaP) is diagnosed and, specifically, the role played by prostate‐specific antigen (PSA), a role that has been vigorously debated ever since its acceptance by the FDA as a monitoring agent in 1985, and similarly as a screening agent in 1994. The following is a brief review of the current status of CaP diagnosis which I trust will widen the reader's perspective of this life‐affecting issue.

Before proceeding, a few things should be made clear: the term PSA is a misnomer, as this protein is not “specific” to the prostate; it has been found in women. Also, PSA is not a tumor marker, as it is produced by both normal and malignant prostate tissues. Because malignant prostate tissue may be more metabolically active than normal tissue, serum PSA levels in men with prostate cancer may be correspondingly higher. (In a nutshell, this is the basis of PSA screening.) However, serum levels of PSA can be elevated by a digital rectal examination (DRE), vigorous exercise, riding a bicycle, and benign prostatic hypertrophy. To further complicate the issue, consider that different methods for measuring PSA can yield different results. Therefore, the odds of a man having prostate cancer based upon a single PSA determination are not much greater than those for any man of his age and race in the general population. Nevertheless, despite these confounding factors, for many years following its introduction an arbitrarily selected serum level of 4 ng/mL was widely accepted by urologists as a threshold for more aggressive diagnostic procedures.

Screening programs based upon PSA yield more false alarms than real fires. Epidemiological studies of comparable groups of PSA‐screened and nonscreened men have shown that, for every life spared in the screened group, 50 men undergo unnecessary treatments. Although high PSA levels may be due to CaP, rarely would a radical treatment be initiated before a biopsy provides direct evidence of the disease.

The most widely accepted metric for assessing the virulence of CaP is the Gleason score. From a series of needle biopsies obtained transrectally under ultrasonic guidance, the pathologist seeks out clusters of malignant cells. Depending upon their degree of malignancy, the two most prevalent clusters are given scores of 1 through 5, and their sum, 2 through 10, is the Gleason score. A score of 6 or less is viewed as low‐grade disease, and the patient may be advised to participate in a program of active surveillance. A score of 7 or higher suggests higher‐grade disease, and treatment is usually recommended. Unfortunately, Gleason scores vary with the skill and predilections of the pathologist. A score of 6 by one pathologist may result in the patient entering an active surveillance program, whereas a score of 7 obtained by another pathologist for the same tissue specimens may result in the patient undergoing a life‐changing treatment.

To further muddy the waters of diagnosis, consider that in studies conducted over the past half‐century, CaP was frequently found in the autopsy specimens of men who died of other causes. A meta‐analysis of these data shows that disease prevalence increases with each decade from roughly 5% for men under 30 years of age to 60% for those over 80. There is no way of knowing which of these men would have died of CaP had they lived longer, but considering that annual death rates over the period 1960 and 2010 ranged between 25 and 40 per 100,000, that number would have been small. These data suggest that most of the cancers in these autopsied men, especially the older men, had become dormant at some point in their development. But dormant or not, had these men been biopsied while alive, their Gleason scores could very well have led to what we know in hindsight to have been unnecessary treatments.

Active surveillance programs are intended to reduce or delay the number of treatments offered to men suspected of harboring prostate cancer. There are no hard and fast rules for active surveillance programs; they are mainly intended for men whose Gleason scores are 6 or lower, and whose DREs or ultrasound scans are ambiguous. PSA levels play an important role in surveillance programs as rising levels over a period of months suggest developing disease. When this happens, biopsies are recommended to determine whether there is a corresponding increase in the patient's Gleason score.

I trust that this letter will spur other physicists who have unique knowledge of, or experience with, an aspect of cancer that would be of interest to other physicists to write similar summaries.

## COPYRIGHT

This work is licensed under a Creative Commons Attribution 3.0 Unported License.

